# A Bilateral Pediculated Palatal Periosteal Connective Tissue Flap for Coverage of Large Bone Grafts in the Anterior Maxillary Region

**Published:** 2012

**Authors:** Amin Rahpeyma, Saeedeh khajeh Ahmadi, Vahid Reza Hosseini, Hamidreza Azimi

**Affiliations:** 1 *Assistant Professor oral and maxillofacial surgery, Oral and Maxillofacial Diseases Research Center, Faculty of dentistry, Mashhad University of Medical Sciences, Mashhad, Iran*; 2 *Assistant Professor oral and maxillofacial pathology, Dental Research Center, Faculty of dentistry, Mashhad University of Medical Sciences, Mashhad, Iran *; 3 **Department of oral and maxillofacial surgery, Dental Research Center, Faculty of dentistry, Mashhad University of Medical Sciences, Mashhad, Iran**

**Keywords:** Bone graft, Reconstructive surgical procedures, Surgical flap

## Abstract

**Introduction:**

Coverage of bone grafts is very important in reconstructive surgery. In edentulous alveolar ridges this coverage is particularly important for supporting dental prostheses. Here we present the case of a patient with a large deficient maxillary anterior region that was reconstructed with a bilateral palatal submucosal periosteal connective tissue flap: a soft tissue reserve for upper jaw reconstructive surgeries. The bilateral pediculated palatal periosteal connective tissue flap was used for coverage of a large bone graft in the anterior maxillary region.

**Conclusion:**

Palatal submucosa can be used as a soft tissue reserve in upper jaw reconstructions.

## Introduction

Bone grafting is used for replacement of hard tissue that has been lost due to trauma or pathological processes that involve the jaw regions. Soft tissue coverage of bone grafts in the alveolar region is an important factor for the success of the graft. Ideally this coverage should meet the following criteria:

1. Provide complete coverage of the bone graft (1,2).

2. Play a role in nourishment of the graft (3,4).

3. Provide histological similarity between the preexisting soft tissue and the new soft tissue coverage (5,6).

4. Have an appropriate volume (not bulky) with no need for reshaping procedures on the soft tissue (7).

5. Maintain the osteogenic capacity of the soft tissue coverage (8).

In this case report with detailed technical notes we present a soft tissue flap created from palatal connective tissue that was used to cover a 3 × 2 cm^2^ bone graft in the anterior maxilla, and simultaneously reconstruct hard and soft tissue in the vertical and horizontal dimensions.

## Case Report

**Fig 1:**
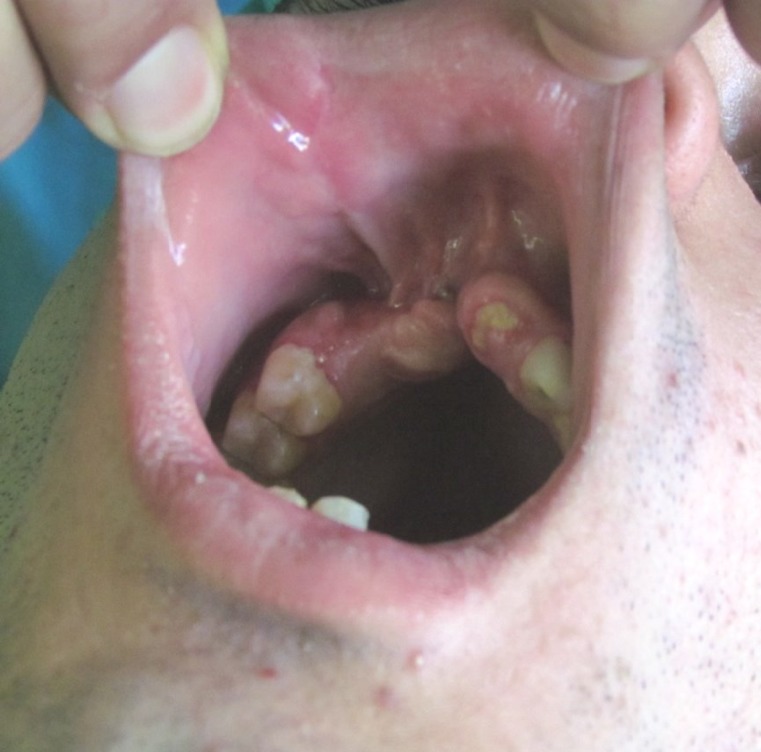
Photograph showing the maxillary anterior alveolar ridge defect

The patient was a 20-year-old male who had experienced a motor vehicle accident three months earlier. After primary care and treatment of facial fractures he was referred for reconstruction of a deficient anterior maxillary ridge. The defect in the anterior maxilla extended from the distal surface of the maxillary left lateral incisor to the mesial surface of the maxillary right second bicuspid. The edentulous ridge was severely deficient in the buccolingual and vertical dimensions. Soft tissue scarring in the vestibule and previous surgeries made reconstruction more difficult (Fig 1).

**Fig 2:**
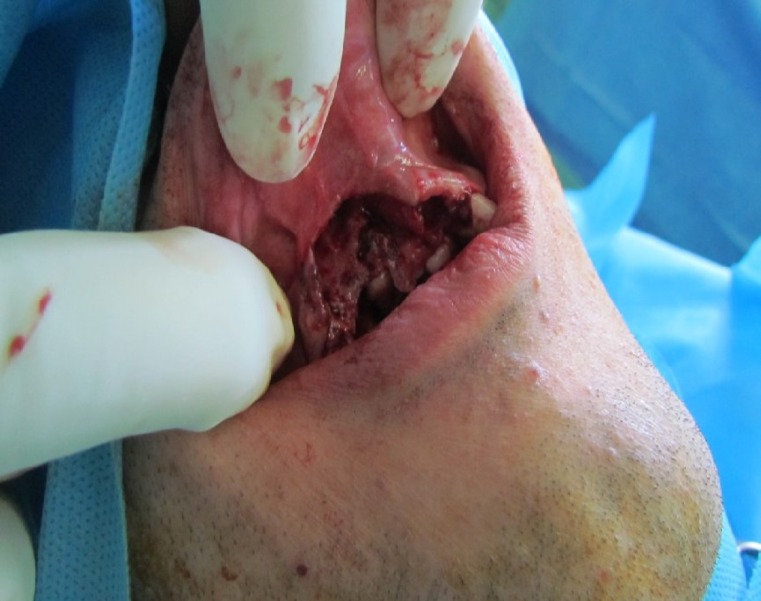
Photograph showing the actual bone defect and soft tissue deficiency after the bed preparation

Surgical procedure:

Under general anesthesia an incision was made on top of the residual crest to access the underlying bone, which was atrophied, thin, and irregular (Fig 2). In order to obtain a pediculated periosteal connective tissue flap from the palate, we injected 2% lidocaine with epinephrine (1:80,000) subperiostealy in the hard palate region to facilitate subperiosteal dissection and control of hemostasis.

The flap needed to have sufficient length and width to reach the vestibular depth and cover the bone graft. So we designed a long incision horizontally in the hard palate with a 5 mm vertical distance from the scalloped gingival margin that reached from the second molar to the ipisilateral incisor. Sharp dissection with a no. 15 blade was used to separate the connective tissue from the overlying epithelium. This dissection continued toward the midline. Great caution was needed to avoid perforation or thinning of the overlying mucosa. Blunt dissection with a periosteum, elevated this tissue from the bone. 

The most distal part of the flap was incised vertically and brought to the anterior maxillary buccal region underneath the mucosa.

Hemorrhage from the greater palatine artery was controlled by cautery and surgicel® applied for dead space management. The palatal mucosal incision was sutured primarily and the whole procedure was done bilaterally in the palate.

**Fig 3:**
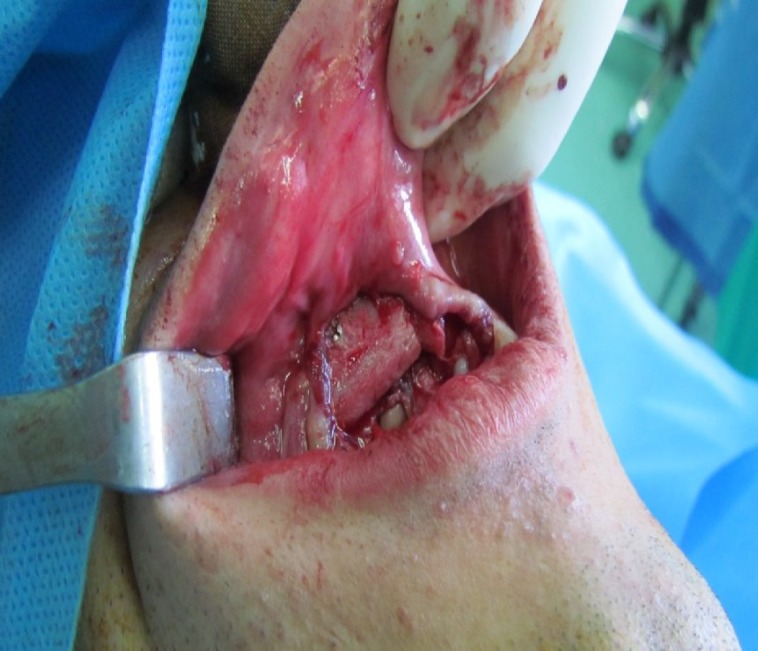
Photograph showing the corticocancellus bone graft fixed in place with a titanium mini screw

A corticocancellus bone graft (3 × 2 cm^2^) was obtained from anterior iliac crest with a medial approach and fixed in the recipient site with a titanium mini screw. The height of the bone graft was 4 mm more than the residual bone and augmented the deficient ridge (Fig 3). 

**Fig 4:**
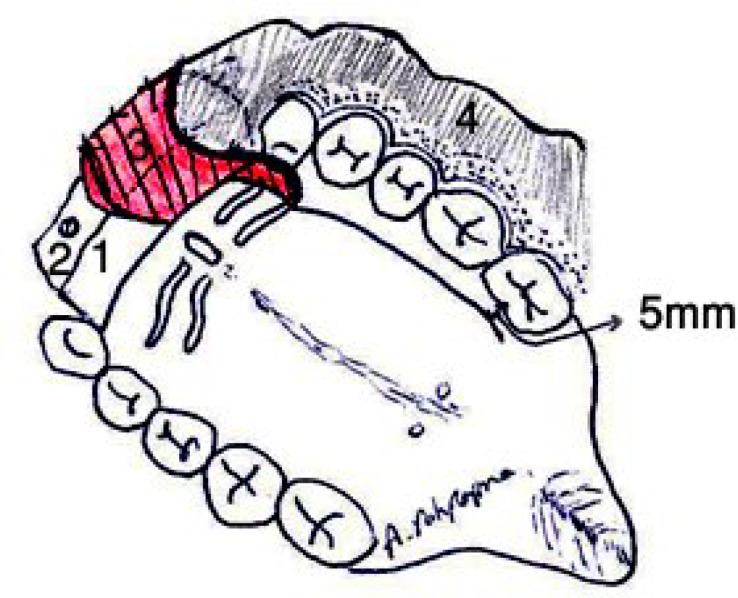
Schematic diagram of the anteriorly based pediculated palatal periosteal connective tissue flap. 1 Bone graft, 2 Titanium screw, 3 Pediculated palatal periosteal connective tissue flap, and 4 Alveolar mucosa

**Fig 5:**
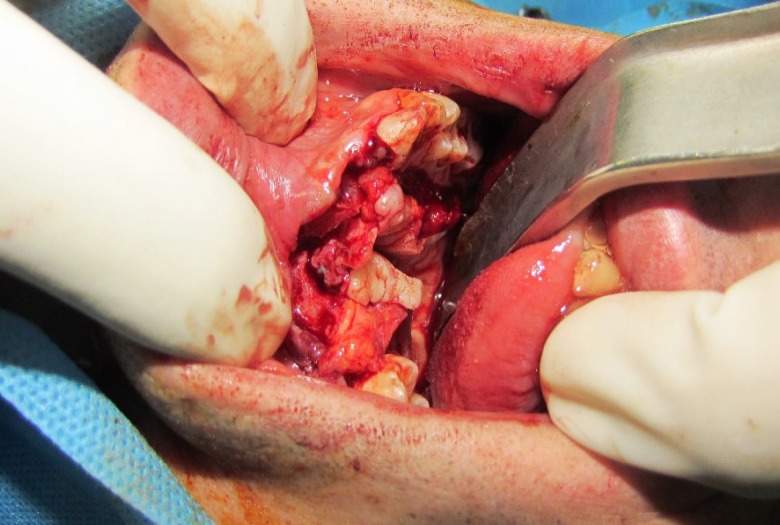
Palatal view of the bilateral pedicle flaps

**Fig 6:**
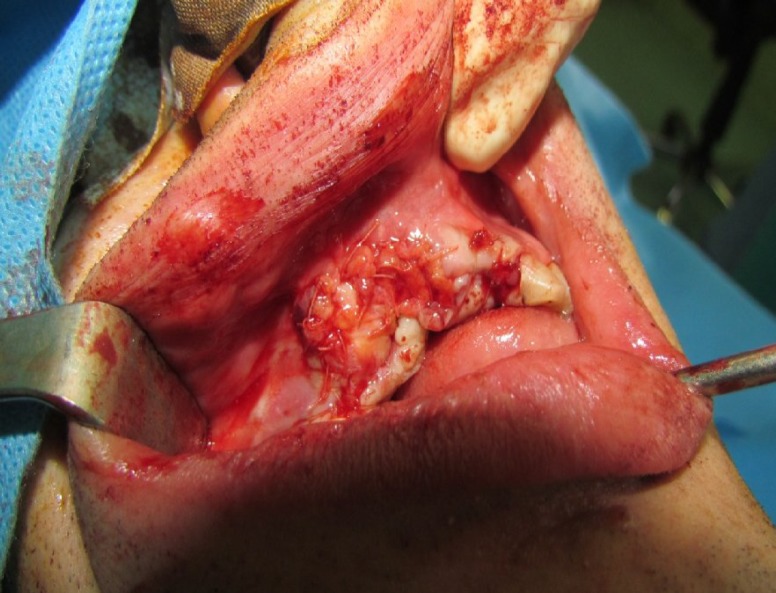
Photograph of the bilateral palatal submucosal periosteal connective tissue flap covering the exposed bone graft

The bone graft was covered with bilateral anteriorly-based soft tissue flaps from the periosteal connective tissue of the hard palate. The edges of the flaps were sutured in the depth of the vestibule and together in the midline. Sutures were made with 4-0, 5-0 vicryl® (Figs 4–6).

## Discussion

The use of a pediculated periosteal connective tissue flap of the hard palate is valuable in maxillary reconstructions (9, 10). The flap has a random pattern blood supply, can have a width/ length ratio of up to 1/5 (8), and is used for minor ridge reconstruction of maxillary esthetic regions and simultaneously reconstructs both hard and soft tissue defects (9, 10). The flap also contains periosteum and connective tissue from the hard palate, allowing the bone graft to be covered with osteogenic tissue. The pedicled blood supply derived from the connective tissue periosteal plexus within the flap provides the biological basis for predictable bone graft coverage. The simultaneous hard and soft tissue reconstruction also reduces the number of operations that are needed for the reconstruction and reduces the time until placement of dental implants. This flap does not need to be covered by mucosa and heals by secondary epithelialization, so the vestibular depth does not decrease, which is an important factor for prosthetic replacement of lost teeth in the most critically esthetic area of the jaw. In this report we have shown that this flap is capable for successfully covering large bone grafts. 

## Conclusion

Palatal submucosa can be used as a soft tissue reserve in upper jaw reconstructions. The donor site is near the surgical field and has minor morbidity. The surgical technique is simple, quick, and predictable. Simultaneous hard and soft tissue augmentation in the most esthetic area of the jaw is possible using this procedure.
